# Effects of maturity stage and mancozeb on phyllosphere microbial communities and the plant health potential of silage maize

**DOI:** 10.3389/fpls.2025.1581401

**Published:** 2025-06-04

**Authors:** Qingbiao Xu, Qiu Yang, Xianli Lu, Shaoshen He, Xueling Ma, Dan Wu, Yuanyan Meng, Liuxing Xu

**Affiliations:** ^1^ College of Agronomy and Life Sciences, Zhaotong University, Zhaotong, China; ^2^ College of Plant Protection, Yunnan Agricultural University, Kunming, China; ^3^ College of Animal Science and Technology, Yunnan Agricultural University, Kunming, China

**Keywords:** mancozeb, phyllosphere microorganisms, plant health, silage maize, stage

## Abstract

Mancozeb is often used to supplement the nutritional requirements of maize for elements such as manganese and zinc, as well as for the control of diseases such as large blotches and stripe blotches. The objective of this study was to assess the effects of different concentrations of mancozeb on phyllosphere microbial diversity and plant health in silage maize. The experimental treatments comprised three maturity stages (big trumpet, milk, and dough) and four mancozeb concentrations (control: CK, equal amounts of distilled water; low concentration, 1500-fold dilution; medium concentration, 1000-fold dilution; and high concentration, 500-fold dilution). The fresh matter yield of silage maize increased by 36.6% and 9.07% in the low and high treatments than in the CK, respectively. Compared with the CK, the application of mancozeb slightly improved the photosynthetic properties of the silage maize. Specifically, compared with CK, the net photosynthetic rate, transpiration rate, and intercellular CO_2_ concentration in the low-concentration treatment increased by 10.4%, 50.2%, and 28.5%, respectively. Compared to the dough stage, the net photosynthetic rates increased by 64.8% and 93.2%in the big trumpet and milk stages, respectively, and transpiration rates increased by 66.4% and 155%, respectively. Total phenols, proline, reducing sugars, vitamin C, free amino acids, and inorganic phosphorus contents were the highest (P < 0.05) in the leaves at the dough stage. The low and medium treatments reduced the relative abundance of the harmful fungus *Epicoccum* compared to CK (high > CK > low > medium), and compared to CK, the low treatment increased *Pantoea*, *Chryseobacterium*, *Microbacterium*, *Massilia*, *Filobasidium*, *Papiliotrema* and other beneficial microorganisms in relative abundance. The relative abundance of *Parasola* was significantly higher (P < 0.05) than that of the CK at low and medium treatments. At the genus level, the fungal community with the highest relative abundance was *Symmetrospora* (high > low > CK > medium). Based on the yield and utilization of silage maize and considering the changes in the diversity of microorganisms attached to the surface of silage maize leaves, this study recommends the use of low concentrations of mancozeb and harvesting at the milk stage.

## Introduction

As an essential forage crop, silage maize is cultivated globally, and its production yield and quality play pivotal roles in the sustained development of the global livestock industry. Silage maize has high dry matter production capacity per unit area, excellent fermentability during storage, and is easily digestible for ruminants (Karnatam et al., 2023). The contents of crude fiber, acid detergent fiber, neutral detergent fiber, and nitrogen-free extract are 23.97%, 27.62%, 51.60%, and 59.68%, respectively (Zhao et al., 2022b). The global silage maize market is projected to grow at a compound annual growth rate of 7.84% from 2021 to 2030 (Karnatam et al., 2023). The yield and quality of silage maize are influenced by a variety of factors, especially crop management practices (Han et al., 2024). Research has found that increasing the nitrogen fertilizer application rate from 0 to 200 kg ha^-1^ can significantly enhance the dry matter yield of maize (Sheaffer et al., 2006). On the other hand, silage maize often encounters various diseases during its growth process, significantly reducing its yield and quality (Mueller et al., 2020). This ultimately affects the value of the feed and the economic benefits for the livestock industry (Reed et al., 2021). For example, maize in the southwestern and northern regions of China is severely challenged by the foliar diseases maize grey spots and big blotches, respectively (Liu et al., 2015). Foliar fungicides are widely used (Lai et al., 2016; Wathaneeyawech et al., 2015), even prophylactically in the absence of an obvious disease (Schneider et al., 2023). The use of foliar fungicides has been shown to reduce both neutral and acid detergent fiber contents in silage maize (Kalebich et al., 2017). Mancozeb is a highly effective foliar fungicide with excellent bactericidal effect and preventive and protective action (Emara et al., 2021). It not only enhances the resilience of maize but also prevents and treats a variety of maize diseases (Munkvold et al., 2001). However, little is known about the mechanisms through which Mancozeb affects the leaf microbiome of silage maize.

In recent years, with the rapid development of molecular biology techniques, an increasing number of researchers have begun to pay attention to the species and relative abundance of microorganisms in the phyllosphere (both inside and on the surface of leaves), providing a more efficient approach to reveal the impact of pesticides on forage phyllosphere microbial communities. Among the microorganisms found in the maize leaf microenvironment, *Alcaligenaceae*, *Erwiniaceae*, and *Pseudomonadaceae* have been the most prominent bacteria discovered (Wu et al., 2023), while fungi dominated by *Cryptococcus*, *Alternaria*, and *Pyrenochaeta* have also been identified (Kong et al., 2020). In addition, *Exserohilum*, *Bipolaris*, *Cercospora*, and *Curvularia* are more likely to be found in highly susceptible maize varieties (Luo et al., 2023). Most physicochemical properties of the leaf surface are closely related to the quantity of bacteria and fungi. For instance, leaf area and chlorophyll content are positively correlated with the number of aerobic bacteria (Tang et al., 2023). Moreover, phosphorus, nitrogen, flavonoid, and tannin content are strongly correlated with fungal operational taxonomic units (OTUs) throughout the leaf (Luo et al., 2023). Previous studies have indicated that foliar fungicides regulate leaf microbiota (Whitaker et al., 2025). However, these studies have primarily focused on pesticide toxicity or its impact on microbial resistance (Qiu and Shi, 2014), neglecting their potential effects on the physiological properties of forage crops or the number and relative abundance of associated microorganisms.

The close relationship between plant health and environmental microorganisms has become a popular research topic. However, research on the types and quantities of epiphytic microorganisms in forage crops remains inadequate, particularly regarding their interactions with pesticides, which has received little in-depth exploration. Previous studies have analyzed the key factors that influence the surface microbiota of forage plants (Tang et al., 2023), highlighting their important roles in microbial migration, growth, and mortality. As a significant source of forage for ruminants, the phyllosphere microbial community of silage maize determines silage fermentation quality. Nevertheless, there are few reports on how forage crops maintain beneficial microbial communities for silage fermentation quality or suppress harmful microorganisms to prevent and control foliar diseases. Therefore, the present study aimed to clarify the influence of different growth stages and concentrations of mancozeb on epiphytic bacteria and fungi in silage maize. The following hypotheses were proposed: 1) the milk stage of silage maize represents the peak in the quantity of net photosynthetic rate; and 2) low concentrations of foliar fungicides not only facilitate yield enhancement but also effectively inhibit the growth of undesirable microorganisms.

## Materials and methods

### Experimental site

The experimental site was located at the experimental field of Zhaotong University (Zhaoyang District, Zhaotong City, Yunnan Province; 27°36′N, 103°74′E; at an altitude of 1989 m). The soil type was lithic humus soil. According to data from the Zhaotong Meteorological Bureau, the average annual temperature over the past 20 years was 12.3°C, with an annual precipitation of 682 mm. During the growing period of silage maize, the total precipitation and average temperature were 608.7 mm and 17.2°C, respectively.

### Experimental design and crop management

The experiment consisted of three maturity stages (big trumpet, milk, and dough stages) and four fungicide concentrations (control: CK, equal amounts of distilled water; low concentration: 1,500 × dilution; medium concentration: 1,000 × dilution; high concentration: 500 × dilution). The fungicide used was mancozeb (Production: Sichuan Runer Technology Co., Ltd.; Formulation: wettable powder, 80% active ingredient content) at 750 g hm^-2^. To reduce the interference of the external environment on the experimental results, this study was conducted as a pot planting experiment. Before the experiment, the soil was thoroughly mixed and packed into foam boxes (inner dimensions — length: 540 mm, width: 385 mm, height: 300 mm). Each pot was filled with 13 kg of soil (natural moisture content: 23%), and the spacing between pots was set at 50 cm. Three replicates per treatment were randomized and arranged in blocks. Fertilizer was applied at a uniform rate of 35 g boxes^-1^ (15:15:15 ratio of N:P_2_O_5_:K_2_O). During culture, fertilizer was applied at a rate of 40% at the seedling stage and 60% at the jointing stage. Two seeds (Zhaohuang 24) were sown 8 cm from each of the four corners of the pots, and the excess seedlings were removed after emergence, leaving one plant in each position (four plants per pot). Four pots were planted in each treatment. The experimental materials were placed in a greenhouse with 85% light transmission and were planted on April 27, 2022, at the experimental base of the College of Agronomy and Life Sciences, Zhaotong College. Samples were collected on August 23 (big trumpet stage), September 18 (milk stage), and October 16 (dough stage) of the same year. Mancozeb was sprayed, with two sprays being applied throughout the reproductive period: at the jointing stage (July 4, 2022) and at the big trumpet stage (August 13, 2022), with 40% applied at the jointing stage and 60% applied at the big trumpet stage. All treatments were irrigated at the same volume during the growth period.

### Sample collection

The experimental materials were sampled at the big trumpet, milk, and dough stages. A sunny morning was selected to determine the net photosynthetic rate, transpiration rate, intercellular CO_2_ concentration, intercellular CO_2_ partial pressure, stomatal conductance of water vapor, total conductance of water vapor, and total conductance of CO_2_ in maize (healthy leaves at the same location were selected) using an LI6800 photosynthesizer (LI-COR, Lincoln, NE, USA). The first leaf under the spike was collected under aseptic conditions, and the material was divided into two parts: one part was used to determine the physiological and chemical properties of the leaf, and the other part was placed in liquid nitrogen for bacterial and fungal community structure analysis.

### Physiological and chemical properties analyses

Photosynthetic parameters were determined using a LI6800 photosynthesizer with the following parameter settings: flow rate of 500 µmol s^-1^, relative humidity of 50%, CO_2_ concentration of 400 ppm, rotational speed of 10,000 rpm, and light intensity of 1,500 µmol^-1^ m^-2^ s^-1^. The method of Bi (Bi et al., 2017) was referred to for determination of cuticle permeability, with slight modifications. That is, the leaves were first darkened to avoid water loss for half an hour to induce stomatal closure of the leaves, and then, after determining the fresh weight of the leaves using a 1-in-10,000 electronic analytical balance, the leaves were placed in an open chamber and naturally dehydrated for 10 h at 25°C, being weighed at 1 h intervals. Finally, the leaves were placed in an oven at 37°C for 72 h of water loss. The resulting weight was recorded as the dry weight of the leaves, while water retention capacity was determined with reference to the method of Ni (Ni et al., 2015). Stomatal on the upper and lower surfaces of the leaves were determined using the nail polish blotting method (Bi et al., 2017).The density of epidermal trichomes on maize leaves was determined using the leaf imprint method (Patel et al., 2021).

Proline content was determined by referring to Pirzad’s method, with slight modifications (Pirzad et al., 2011). The samples were ground into a homogenate with 80% ethanol, extracted for l h under darkness, filtered, and subsequently analyzed using the ninhydrin method. Total phenol content was determined by referring to Beyhan Ö’s method (Beyhan et al., 2010), the samples were water-bathed above 80°C for 40 min, followed by the addition of 5 ml of folin’s reagent for 3 min after shaking, and finally measured at 700 nm on a UV spectrophotometer. Soluble protein content was determined using thomas brilliant blue G-250 staining solution (Zhang et al., 2015). The reducing sugar content was determined at 540 nm using the 3,5-dinitrosalicylic acid method (Toma and Leung, 1987). The samples were ground to pulp using an oxalic acid-EDTA solution, followed by the addition of oxalic acid-EDTA, metaphosphoric acid-acetic acid, 5% sulfuric acid, and 5% ammonium molybdate; the absorbance values were determined at 700 nm (Liu et al., 2014). Free amino acid content was determined using the ninhydrin method, and the absorbance value of the samples was determined at 570 nm. Inorganic phosphorus content was determined using the phosphomolybdenum blue colorimetric method, the sample leaves were homogenized by mortar and pestle, and the absorbance was determined at 650 nm in a water bath at 45°C for 25 min.

### Microbial community analysis

Total microbial community DNA was extracted using an EZNAPlant DNA kit (Omega Bio-tek, Norcross, GA, USA) according to the manufacturer’s instructions. DNA was extracted using 1% agarose gel electrophoresis, and DNA concentration and purity were determined using a NanoDrop 2000 spectrophotometer. To address the common issue of sequence similarity between plant endophytic bacteria and chloroplast or mitochondrial rRNA coding sequences, a nested polymerase chain reaction (PCR) assay was used. The V5–V7 regions of the 16S rRNA and ITS genes of the strains were amplified with primers 779F (5′AACMGGATTAGATACCCKG-3′) and 1193R (5′-ACGTCATCCCCACCTTCC-3’) using the thermocycler PCR system (GeneAmp 9700, ABI, USA). PCR products from each same sample were mixed and resolved on a 2% agarose gel. The recovered DNA fragments were purified using an AxyPrep DNA Gel Extraction Kit (Axygen Biosciences, Union City, CA, USA) according to the manufacturer’s instructions, and their concentrations were quantified using a Quantus Fluorometer (Axygen Biosciences, Union City, CA, USA). Library construction of the purified PCR products was performed using a NEXTFLEX Rapid DNA-Sequencing Kit. Sequencing was performed using Illumina’s PE300 platform. Quality control and sequence assembly were performed using Fastp software (OriginPro^®^2021b, OriginLab Corp., WA, USA).

### Statistical analysis

In order to determine the effects of maturity stage and mancozeb concentration on the physiological properties and structural microbial diversity of silage maize leaves, the data were analyzed using analysis of variance, which was conducted on SPSS 27.0. In addition, to investigate the linkages among indicators (all variables measured), correlations among samples were analyzed based on the relative abundance of microorganisms (genera) and environmental factors. The data in the tables are presented as mean ± standard error of three experiments.

## Results

### Effects of mancozeb concentration and maturity stage on silage maize yield and leaf physiological properties

Mancozeb concentration had a significant effect (P < 0.05) on silage maize yield (**Supplementary Figure S1**). Although there was no difference between the medium, high, and CK treatments, they were all significantly lower (P < 0.05) than the low treatment. Specifically, the fresh matter yield of maize increased by 36.6% and 9.07% in the low and high treatments, respectively, but was 28.8% lower in the medium treatment than in the CK.

The maturity stage had a significant effect (P < 0.01) on the physiological properties of the silage maize (**Table 1**). Compared to the dough stage, the net photosynthetic rate increased by 64.8% and 96.5% at the big trumpet and milk stages, respectively, the transpiration rate increased by 66.5% and 155%, total conductivity of water vapor increased by 75.8% and 188%, and the total conductivity of carbon dioxide increased by 75.9% and 190% Compared with the CK, the application of mancozeb slightly improved the photosynthetic properties of the silage maize. It also improved in the water retention, which increased by 14.8, 18.1, and 13.9% in the low, medium, and high treatments, respectively. The interaction between the maturity stage and mancozeb concentration had a significant effect (P < 0.01) on water vapor pore conductance and water retention. In conclusion, the physiological properties were higher at the milk stage than at the big trumpet and dough stages, and the photosynthetic properties were higher at low treatment than at the CK, medium, and high treatments.

**Table 1:** Effects of maturity stage and mancozeb concentration on physiological properties of silage corn leaves (n = 12).

Maturity stage and treatment		Net photosynthetic rate (µmol m^-2^ s^-1^)	Transpiration rate (mmol m^-2^ s^-1^)	Intercellular carbon dioxide concentration (µmol mol^-1^)	Pore conductivity of water vapor (mmol m^-2^ s^-1^)	Total conductivity of water vapor (mmol m^-2^ s^-1^)	Total conductivity of carbon dioxide (mmol m^-2^ s^-1^)	Moisture retention capacity (%)
Maturity stage (MS)	Big trumpet stage	23.4a	2.63b	80.5b	126c	122b	76.5b	47.2b
	Milk stage	27.9a	4.03a	141a	210a	200a	126a	63.6a
	Dough stage	14.2b	1.58c	83.7b	70.5c	69.4c	43.5c	49.1b
	Average value	22.1	2.79	103	135	130	82	53.3
	Quadratic sum	1102	34.8	26302	118019	103590	41327	1933
	*F*	17.5	23.9	6.26	22.3	22.6	22.6	9.214
Mancozeb concentration (MC)	CK	20.2	2.11	88.7	109	106	66.6	41.6
	Low	22.3	3.17	114	156	150	94.1	56.4
	Medium	23.5	3.01	99.8	133	128	80.7	59.7
	High	22.6	2.8	110	143	138	86.5	55.5
	Average value	22.1	2.79	104	135	130	82.0	53.3
	Quadratic sum	56.0	5.35	3242	10676	9230	3660	1734
	*F*	0.297	1.03	0.36	0.59	0.58	0.58	5.05
*P* value	MS	0.000	0.000	0.005	0.000	0.000	0.000	0.001
	MC	0.828	0.394	0.779	0.629	0.633	0.635	0.06
	MS×MC	0.691	0.320	0.001	0.085	0.091	0.091	0.000
	Quadratic sum (MS×PE)	125	4.43	38031	26738	22917	9128	1141
	*F* (MS×MC)	0.65	1.25	6.31	2.15	2.1	2.1	7.77
SEM		1.30	0.23	9.20	12.8	11.9	7.53	2.07

Different lowercase letters in the same column represent significant difference between maturity stage or mancozeb concentration (P < 0.05). SEM, standard errors of the mean.

**Figure 1:**
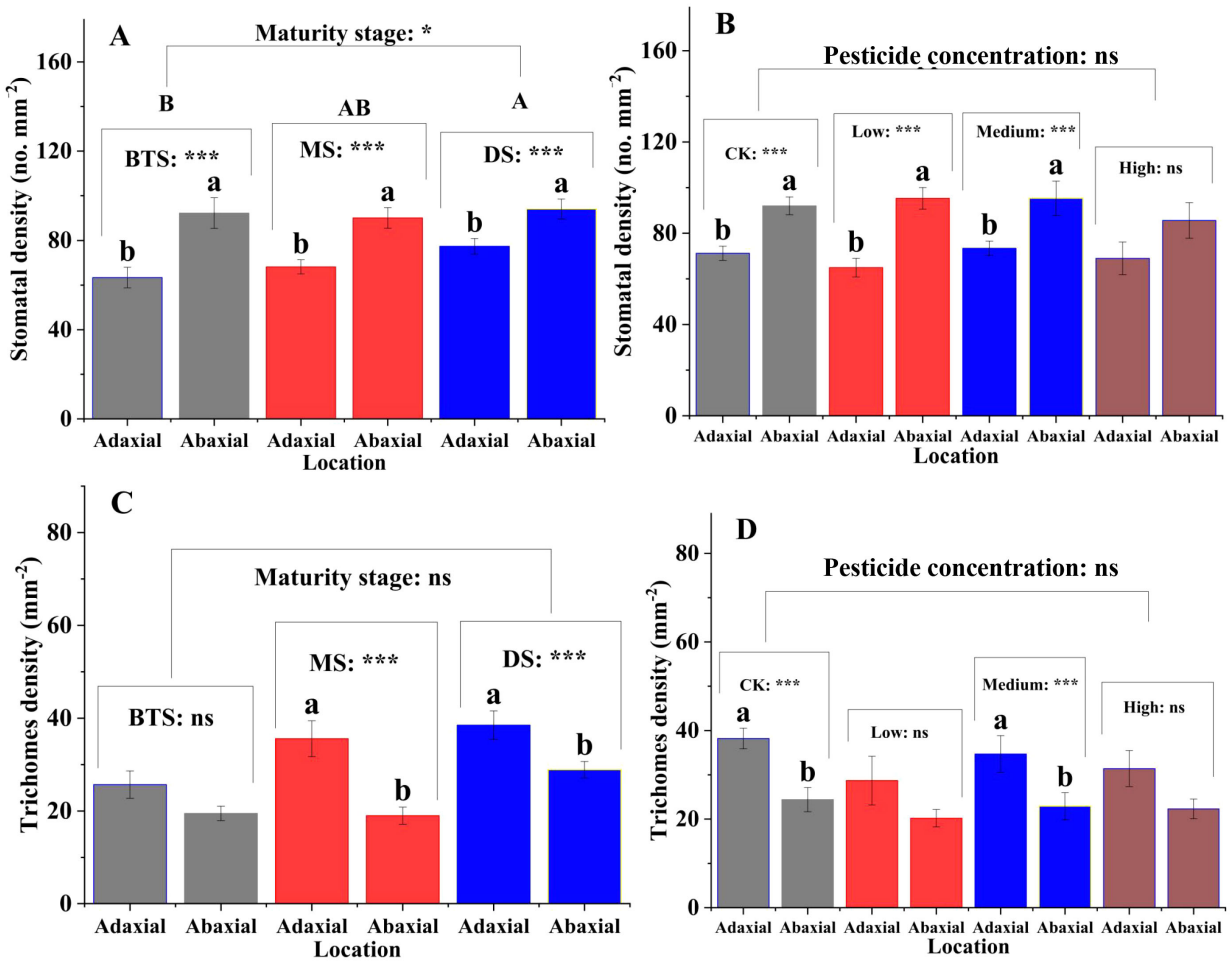
Effects of maturity stage and pesticide concentration on stomatal and trichomes densities of silage corn leaves. Lowercase letters indicate significant differences between different locations of the maturity stage or mancozeb concentration (P < 0.05). **(A)** Lowercase letters indicate significant differences in stomatal density on leaf surfaces at different positions (P < 0.001), while uppercase letters indicate significant differences in stomatal density on leaf surfaces at different maturity stages (P < 0.05); **(B)** Lowercase letters indicate significant differences in stomatal density on leaf surfaces at different positions (P < 0.001); **(C)** Lowercase letters indicate significant differences in trichome density on leaf surfaces at different positions (P < 0.001); **(D)** Lowercase letters indicate significant differences in trichome density on leaf surfaces at different positions (P < 0.001). Error bars represent standard errors. Asterisks indicate significant differences at P < 0.05 (), and *P < 0.001* (**) ; NS, not significant. BTS, big trumpet stage; MS, milk stage; DS, dough stage.

**Figure 2:**
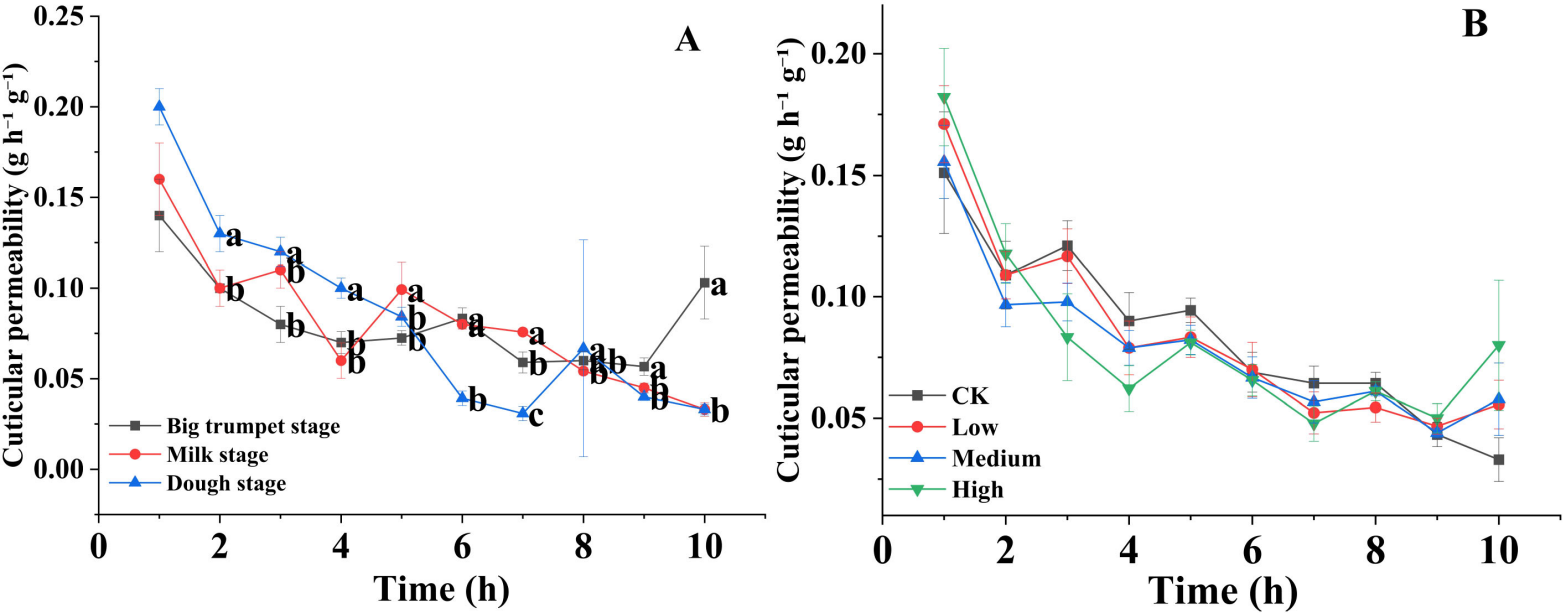
Effects of maturity stage **(A)** and pesticide concentration **(B)** on the cuticular permeability of silage corn leaves. Error bars represent standard errors. Lowercase letters indicate significant differences between mancozeb concentration (P < 0.05).

The stomatal densities on the upper surface of the silage maize leaves were significantly lower than those on the lower surface (P < 0.01) at the big trumpet, milk, and dough stages (**Figure 1A**). The results of different mancozeb concentration treatments were similar (**Figure 1B**). In contrast, trichomes density was higher on the upper surface of the leaves than on the lower surface, which was not affected by maturity stage or mancozeb concentration (**Figures 1C, D**). Regarding cuticle permeability, there was a decreasing trend in silage maize leaf permeability from 1 to 10 h, and among the three maturity stages, cuticle permeability was higher at the dough stage than at the big trumpet and milk stages (**Figure 2A**). The cuticle permeability of silage maize leaves also tended to decrease from 1 h to 10 h, even though the concentration of mancozeb had no significant effect on silage maize leaf permeability (**Figure 2B**).

### Effect of maturity stage and mancozeb concentration on the chemical properties of silage maize leaves

Maturity stage had a significant effect (P < 0.01) on the chemical properties of silage maize leaves (**Table 2**). The contents of total phenols, reducing sugars, and vitamin C in silage maize leaves tended to increase from the big trumpet stage to the dough stage. Proline, reducing sugars, vitamin C, free amino acids, and inorganic phosphorus contents were significantly (P < 0.05) higher in silage maize leaves at the dough stage than at the big trumpet and milk stages. Although there was no significant difference (P > 0.05) in total phenol content between the milk and dough stages, they increased by 63.1% and 96.6%, respectively, compared with the big trumpet stage. Furthermore, there was no significant effect (P > 0.05) of mancozeb concentration on the chemical properties of silage maize, although the vitamin C, soluble protein, free amino acid, and inorganic phosphorus contents were slightly higher in the low treatment than in the CK, medium, and high treatments.

**Table 2:** Effects of maturity stage and mancozeb concentration on chemical properties of silage corn leaves (n = 12).

Maturity stage and treatment		Total phenolic (g kg^-1^)	Proline (ug g^-1^)	Reducing sugar (%, FM)	Vitamin C (mg kg^-1^)	Soluble protein (mg g^-1^)	Free amino acid (ug g^-1^)	Phosphorus (mg g^-1^)
Maturity stage (MS)	Big trumpet stage	0.87b	90.5b	0.73b	17.3b	13.5a	1.51b	0.46b
	Milk stage	1.42a	73.9b	0.73b	26.4b	2.82b	0.79c	0.38b
	Dough stage	1.71a	175a	2.92a	57.5a	4.19b	2.74a	1.06a
	Average value	1.33	113	1.46	33.7	6.84	1.68	0.63
	Quadratic sum	4.31	69985	38.3	10699	815	23.5	3.29
	*F*	16.4	20.4	128	34.6	52.7	50.4	144
Mancozeb concentration (MC)	CK	1.57	102	1.67	35.1	7.65	1.61	0.65
	Low	1.29	111	1.38	35.8	7.98	1.91	0.66
	Medium	1.33	107	1.31	31.9	5.29	1.68	0.58
	High	1.13	132	1.48	32.0	6.45	1.51	0.64
	Average value	1.33	113	1.46	33.7	6.84	1.68	0.63
	Quadratic sum	0.86	4552	0.65	118	40.6	0.79	0.03
	*F*	1.18	0.4	16	0.08	0.42	0.28	0.09
*P* value	MS	0.000	0.000	0.000	0.000	0.000	0.000	0.000
	MC	0.333	0.755	0.921	0.97	0.74	0.84	0.964
	MS×MC	0.559	0.369	0.114	0.122	0.129	0.427	0.142
	Quadratic sum (MS×PE)	0.599	11556	1.40	1605	68.1	1.42	0.11
	*F* (MS×MC)	0.83	1.14	1.94	1.90	1.86	1.04	1.80
SEM		0.08	10.0	0.19	3.54	0.92	0.16	0.05

Different lowercase letters in the same column represent significant difference between maturity stage or mancozeb concentration (P < 0.05). SEM, standard errors of the mean.

### Effect of mancozeb concentration on bacterial and fungal communities

**Figure 3:**
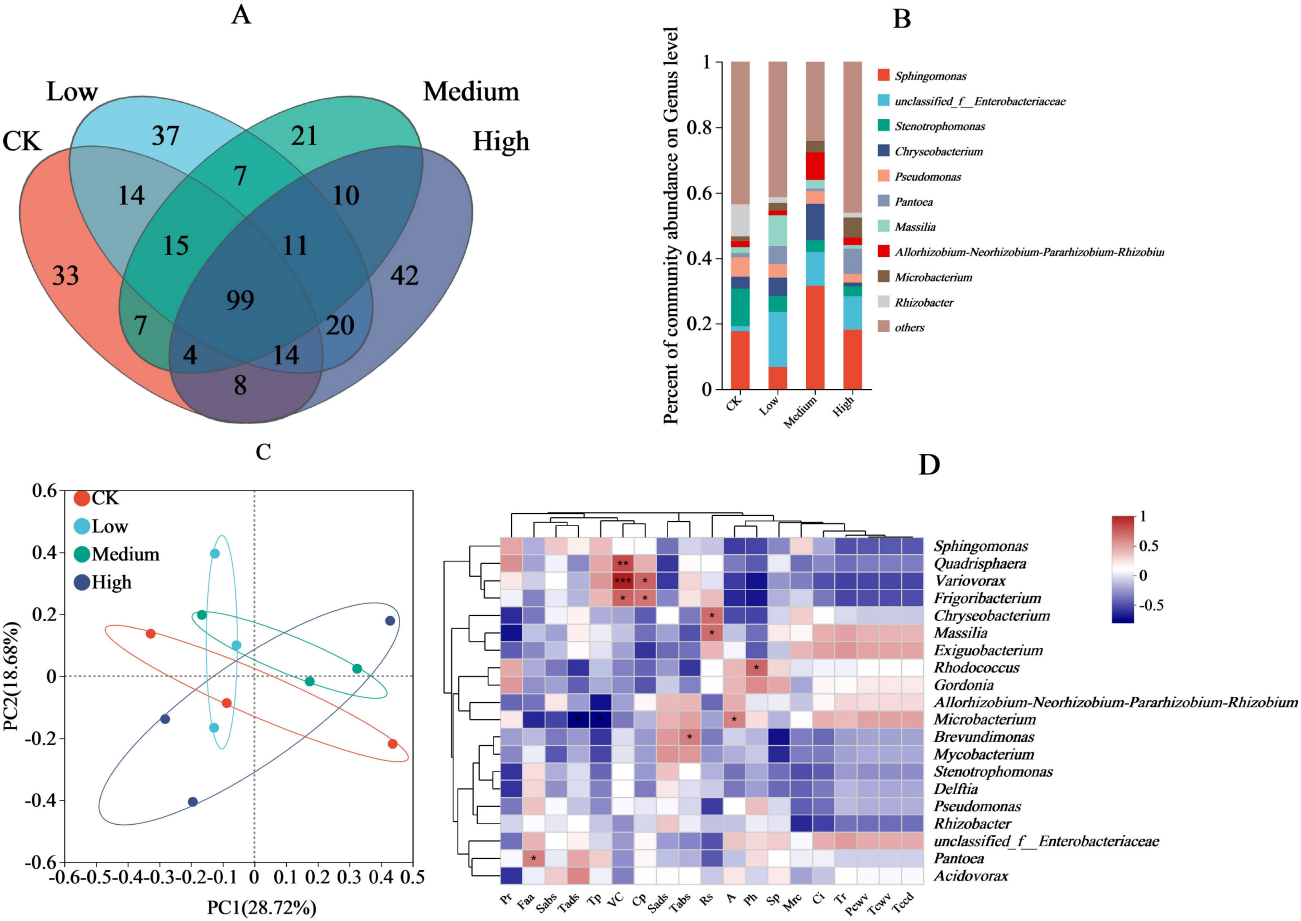
Effect of pesticide concentration on bacterial community (genus). **(A)** different colors represent different mancozeb concentration, overlapping numbers represent the number of species shared by multiple treatments, and non overlapping numbers represent the number of species unique to the corresponding treatment. **(B)** the X-axis indicates the mancozeb concentration treatments, the y-axis indicates the relative abundances of bacterial genera. **(C)** the first axis accounts for 28.72% of the total variance and the second for 18.68%. The original attributes, with their vectors intersecting at (0, 0), are also inserted. The length of each attribute vector is proportional to its contribution to the principal component axis. The ellipse indicates 95% confidence. **(D)** The X-axis and Y-axis represent environmental factors and species, respectively, and the correlation R and P values were obtained through calculation. The R value was displayed in different colors in the graph. * and *** represent P<0.05 and 0.001, respectively. A, net photosynthetic rate; Tr, transpiration rate; Ci, intercellular carbon dioxide concentration; Pcwv, pore conductivity of water vapor; Tcwv, total conductivity of water vapor; Tccd, total conductivity of carbon dioxide; Tp, total phenolic; Pr, proline; Rs, reducing sugar; VC, vitamin C; Cp, cuticular permeability; Mrc, moisture retention; Sads, stomatal density on the adaxial surfaces; Sabs, stomatal density on the abaxial surfaces; Tads, trichomes density on the adaxial surfaces; Tabs, trichomes density on the abaxial surfaces; Sp, soluble protein; Faa, free amino acid; Ph, phosphorus.

In the bacterial community structure, 194, 217, 163, and 208 OTUs were observed in the CK, low, medium, and high treatments, respectively. Of these, the CK shared 142, 125, and 125 OTUs with the low, medium, and high treatments, respectively, whereas the low concentration shared 132 and 144 OTUs with the medium and high treatments, respectively, and the medium and high treatments shared 120 OTUs; 99 OTUs were shared among all four treatments (**Figure 3A**). At the genus level, the bacterial communities with the highest relative abundance were *Sphingomonas* (medium > high > CK > low), *Stenotrophomonas* (CK > low > high > medium), *Chryseobacterium* (medium > low > CK > high), and *Pseudomonas* (CK > low > high > medium) (**Figure 3B**). Principal components 1 and 2 accounted for 18.68% and 28.72% of variance, respectively (**Figure 3C**). At the 95% confidence interval, there was a high overlap between the CK and the low and high treatments, which were clearly separated from the medium treatment. In addition, the low, medium, and high treatments showed a high overlap. Among the environmental factors (**Figure 3D**), the free amino acid content was positively correlated (P < 0.05) with *Pantoea* relative abundance, and vitamin C content was positively correlated (P < 0.05) with the relative abundances of *Frigoribacterium*, *Variovorax* and *Quadrisphaera.* Cuticular permeability was also positively correlated with the relative abundance of *Variovorax* and *Frigoribacterium* (P < 0.05); reducing sugars content was positively correlated with the relative abundance of both *Chryseobacterium* and *Massilia* (P < 0.05); and net photosynthetic rate was positively correlated with the relative abundance of *Micorbacterium* (P < 0.05); inorganic phosphorus content and the relative abundance of *Rhodococcus* was positively correlated (P < 0.05).

**Figure 4:**
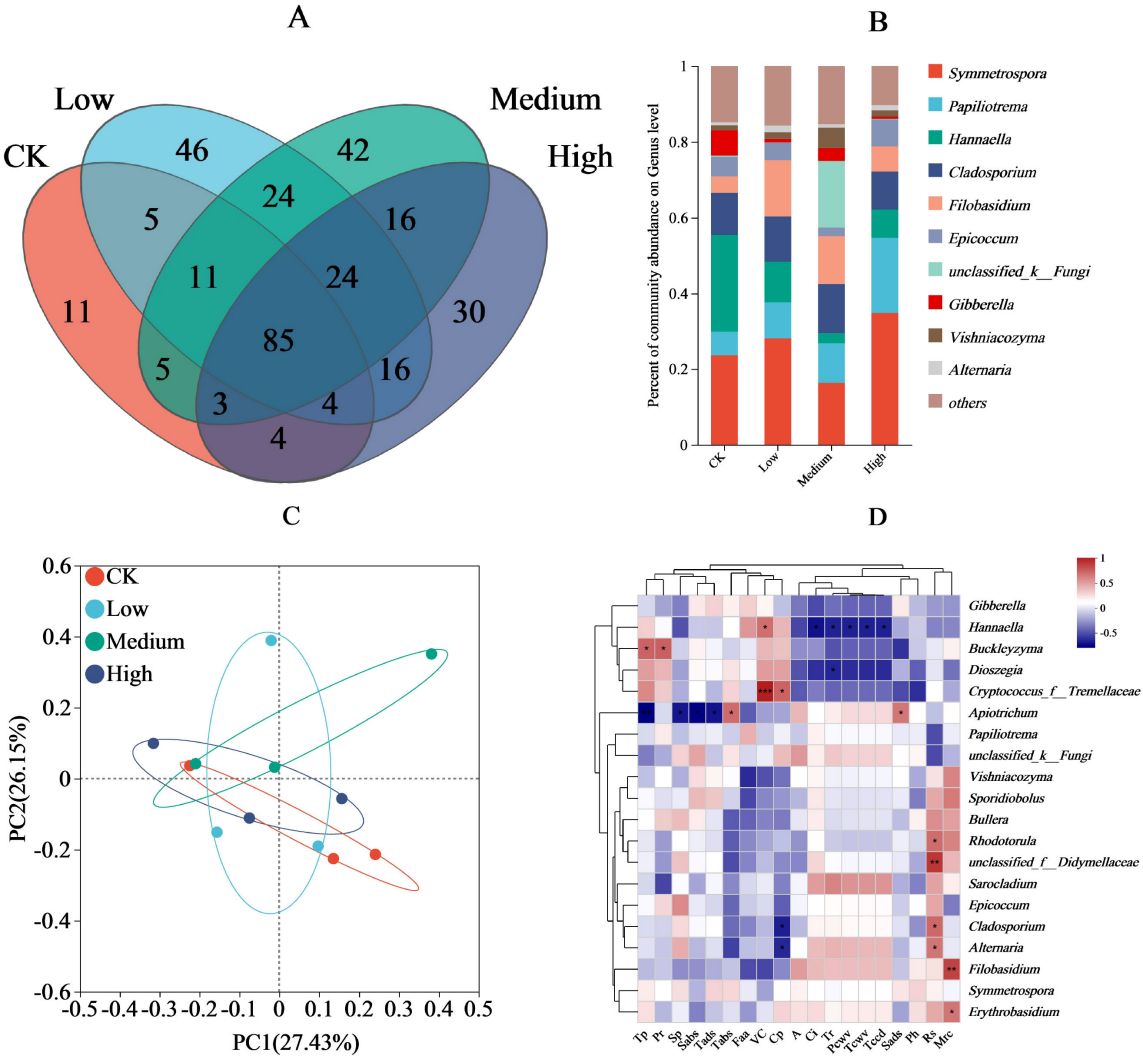
Effect of pesticide concentration on fungi community (genus). **(A)** different colors represent different mancozeb concentration, overlapping numbers represent the number of species shared by multiple treatments, and non overlapping numbers represent the number of species unique to the corresponding treatment. **(B)** the X-axis indicates the mancozeb concentration treatments, the y-axis indicates the relative abundances of fungi genera. **(C)** the first axis accounts for 28.72% of the total variance and the second for 18.68%. The original attributes, with their vectors intersecting at (0, 0), are also inserted. The length of each attribute vector is proportional to its contribution to the principal component axis. The ellipse indicates 95% confidence. **(D)** The X-axis and Y-axis represent environmental factors and species, respectively, and the correlation R and P values were obtained through calculation. The R value was displayed in different colors in the graph. * and *** represent P<0.05 and 0.001, respectively. A, net photosynthetic rate; Tr, transpiration rate; Ci, intercellular carbon dioxide concentration; Pcwv, pore conductivity of water vapor; Tcwv, total conductivity of water vapor; Tccd, total conductivity of carbon dioxide; Tp, total phenolic; Pr, proline; Rs, reducing sugar; VC, vitamin C; Cp, cuticular permeability; Mrc, moisture retention; Sads, stomatal density on the adaxial surfaces; Sabs, stomatal density on the abaxial surfaces; Tads, trichomes density on the adaxial surfaces; Tabs, trichomes density on the abaxial surfaces; Sp, soluble protein; Faa, free amino acid; Ph, phosphorus.

In the fungal community structure, 128, 215, 210, and 182 different OTUs were observed in the CK, low, medium, and high treatments, respectively, of which the CK shared 105, 104, and 96 OTUs with low, medium, and high treatments, respectively; the low, medium, and high treatments shared 144 and 129 OTUs, respectively; the medium and high treatments shared 128 OTUs; and the four treatments shared 85 OTUs (**Figure 4A**). The fungal communities with the highest relative abundance were *Symmetrospora* (high > low > CK > medium), *Papiliotrema* (high > medium > low > CK), *Hannaella* (CK > low > high > medium), *Cladosporium* (medium > low > CK > high), *Filobasidium* (low > medium > high > CK) (**Figure 4B**). Principal components 1 and 2 explained 27.43% and 26.15% of the variance, respectively, and their cumulative contributions were 53.58%. Within the 95% confidence interval, CK had a high overlap with the low and high treatments and a low overlap with the medium treatment (**Figure 4C**). Among the environmental factors (**Figure 4D**), the relative abundance of *Buckleyzyma* was positively correlated with total phenol and proline contents (P < 0.05), the density of trichomes on the abaxial side of the leaf was positively correlated with the relative abundance of *Apiotrichum* (P < 0.05), and the relative abundance of *Hannaella* was positively correlated with relative vitamin C content (P < 0.05). In addition, cuticle permeability was positively correlated with relative abundance of *tremellaceae* (P < 0.05), stomatal pore density on the upper surface was significantly and positively correlated with the relative abundance of *Apiotrichum* (P < 0.05), and reducing sugars content was positively correlated with *Alternaria*, *Cladosporium*, and *Rhodotorula* (P < 0.05). Water retention and *Erythrobasidium* relative abundance was positively correlated (P < 0.05), and water retention and *Filobasidium* relative abundance was positively correlated (P < 0.01). Finally, reducing sugars content and *Didymellaceae* relative abundance was positively correlated (P < 0.01), as were vitamin C content and *Tremellaceae* relative abundance (P < 0.01).

Mancozeb treatment did significantly (P < 0.05) reduce the relative abundance of bacteria (*Comamonadaceae*) (**Table 3**). Among the CK, low, medium, and high treatments, the highest relative abundance of fungi such as *Paramicrothyrium*, *Leptospora*, *Penicillium*, and *Agrocybe* was observed under the low concentration treatment, while the highest relative abundance of fungi such as *Neocoleroa* and *Parasola* was observed under the medium concentration treatment.

**Table 3:** The significant effect of mancozeb concentration on the relative abundance of bacteria and fungi (n=9).

Treatment	Bacteria mean proportion (%)	Fungi mean proportion (%)					
	*Comamonadaceae*	*Neocoleroa*	*Paramicrothyrium*	*Parasola*	*Leptospora*	*Penicillium*	*Agrocybe*
CK	25.2a	0.000b	0.000c	0.000c	0.000d	0.003b	0.000c
Low	7.43b	0.000b	0.013a	0.004b	0.091a	0.020a	0.025a
Medium	3.35b	0.005a	0.000c	0.033a	0.036b	0.003b	0.012b
High	3.53b	0.000b	0.002b	0.000c	0.007c	0.000c	0.000c
*P* value	0.049	0.013	0.025	0.033	0.037	0.044	0.046

Different lowercase letters in the same column represent significant difference between mancozeb concentration (P < 0.05). SEM, standard errors of the mean.

### Effect of mancozeb concentration on normalized shuffle test index of bacterial and fungal communities

**Figure 5:**
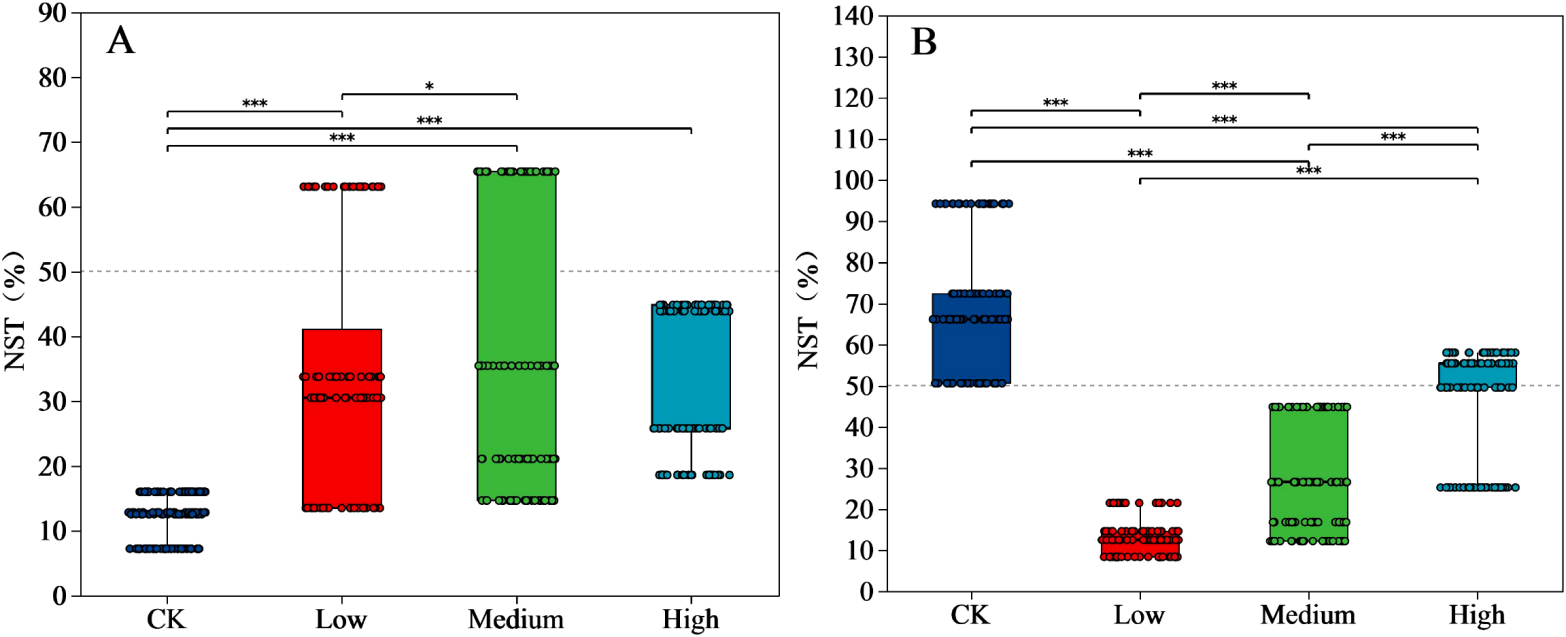
Significance and normalized shuffle test index of bacteria **(A)** and fungi **(B)** relative abundance analyses of different pesticide concentration (genus). Y-axis indicates the normalized shuffle test index, X-axis indicates the mancozeb concentration; The dotted lines were thresholds for deterministic and random divisions, demonstrating significance markers for between-group analysis of variance. * P<0.05, *** P<0.001. NST, normalized shuffle test index.

The normalized shuffle test (NST) index of the bacterial community in the CK was significantly lower (P < 0.05) than those of the low, medium, and high treatments (**Figure 5A**). The NST index tended to increase as the mancozeb concentration increased (Except for high treatment). The NST index of CK fungi was significantly higher (P < 0.05) than the three mancozeb concentration treatments. The NST index of fungi significantly increased (P < 0.05) at medium and high treatments compared to that of the low treatment (**Figure 5B**).

### Effect of mancozeb concentration and plant health-related microorganisms

The relative abundance of the harmful fungus *Epicoccum* was higher in the CK and high treatments than in the low and medium treatments (**Figure 4B**). In the bacterial fraction, *Sphingomonas* was an important plant pathogenic fungal antagonist and promotes plant growth through the production of phytohormones, the effects of which were best demonstrated under the medium concentration treatment conditions (medium > high > CK > low). Similarly, bacteria such as *Microbacterium* and *Pantoea* showed more pronounced promotion effects in the high treatment through enrichment of inter-root microorganisms and activation of defense mechanisms, respectively, whereas beneficial bacteria such as *Massilia* and *Chryseobacterium* demonstrated plant growth promotion advantages under low and medium treatments, while their effects were diminished in the high treatment. In this study, fungi showed functional diversity among the different species. *Cladosporium* caused disease in plants mainly in medium treatment. *Filobasidium* was the most typical plant growth-promoting fungus, with a significant effect in the low treatment (low > medium > high > CK). In contrast, some fungi, such as *Gibberella* and *Epicoccum*, showed pathogenic properties, especially under the CK conditions (**Table 4**).

**Table 4:** Effect of microorganisms on plant health.

Microorganisms	Species	Functionality	Arrange in order
Bacteria	*Sphingomonas*	Bacterial antagonist of plant pathogenic fungi (White et al., 1996) . Promotes plant growth by producing plant growth hormone (Lombardino et al., 2022; Luo et al., 2019).	Medium > High > CK > Low
	*Quadrisphaera*	Plant beneficial bacterial genera associated with resistant strains (Singh et al., 2024).	–
	*Variovorax*	Protect plants from root growth inhibition caused by Arthrobacter spp. and from drought-induced root growth inhibition (Qi et al., 2022).	–
	*Exiguobacterium*	positively affects the formation of plant root hairs (Muvingi et al., 2023).	–
	*Rhodococcus*	Improving plant disease resistance promotes plant growth by synthesizing growth-stimulating phytohormones and inhibiting pathogenic microorganisms (Sun et al., 2024).	–
	*Pseudomonas*	synthesize phytohormones and improve host stress tolerance (Chen et al., 2022).	CK > low = medium > high
	*Mycobacterium*	Involved in autotrophic carbon fixation and antibiotic resistance, they are important antibiotic producers (Li et al., 2014).	–
	*Pantoea*	Beneficial to plant inter-roots (Muvingi et al., 2023).	High > Low > CK > Medium
	*Chryseobacterium*	secretion of plant growth-promoting substances (Kumar et al., 2021).	Medium > Low > CK > High
	*Microbacterium*	promotes the enrichment of specific inter-root microbial taxa and helps the plant to resist adversity (Berendsen et al., 2018).	High > Medium > Low > CK
	*Stenotrophomonas*	activate the plant defense system (Berendsen et al., 2018).	CK > low > medium = high
	*Brevundimonas*	has the potential to improve potato growth and stimulate nitrogen uptake (Naqqash et al., 2020).	–
	*Acidovorax.*	produce secondary metabolites and hormones that promote plant growth (Siani et al., 2021).	–
	*Massilia*	It can be used to control plant pathogenic fungi (Holochová et al., 2020) and has the ability to suppress diseases (Raths et al., 2020).	Low > Medium > CK > High
	*Delftia*	plant growth-promoting bacteria (Morel et al., 2015).	–
	*Comamonadaceae*	Associated with growth promotion and antifungal properties (Unger et al., 2024).	CK > low > high > medium
Fungi	*Alternaria*	pathogenic pathogen (Singh and Goodwin, 2022).	Low > High > CK > Medium
	*Sarocladium*	provide benefits to host plants against biotic and abiotic stresses (Anjos et al., 2020).	–
	*Sarocladium*	Plant pathogens that cause rice grain rot (Ou et al., 2020).	–
	*Cladosporium*	pathogen of leaf spot and other lesions (Bensch et al., 2012).	Medium > Low > CK > High
	*Erythrobasidium*	antifungal activity or growth promoting activity (Khwantongyim et al., 2021; Lu et al., 2024).	–
	*Filobasidium*	Can live permanently on plant hosts and act as survival decomposers altering their lifestyles (Khwantongyim et al., 2021).	Low > Medium > High > CK
	*Epicoccum*	can cause plant diseases (Taguiam et al., 2021).	High > CK > Low > Medium
	*Vishniacozyma*	Induces increased plant resistance (Khwantongyim et al., 2021).	Medium > CK = Low = High
	*Buckleyzyma*	can live permanently on plant hosts and act as survival decomposers altering their lifestyles (Khwantongyim et al., 2021).	–
	*Bullera*	Antifungal activity or growth-promoting activity (Khwantongyim et al., 2021).	–
	*Dioszegia*	Antifungal activity or growth-promoting activity (Khwantongyim et al., 2021).	–
	*Hannaella*	Increase the activity of resistance-related enzymes in apples and prevent blue mold rot (Yang et al., 2023).	CK > low > high > medium
	*Gibberella.*	plant pathogens (Desjardins, 2003).	CK>Medium>Low>High
	*Papiliotrema*	promotes plant growth (Liu et al., 2024) and can be used as a biocontrol agent (Palmieri et al., 2021).	High > Medium > Low > CK

## Discussion

### Effects of mancozeb concentration and maturity stage on yield, leaf physiological and structural properties of silage maize

In general, the milk stage is critical for photosynthesis and the accumulation of organic matter in maize. The full expansion of maize leaves during the milk stage increases the photosynthetic area (Zhao et al., 2022a) and results in a high efficiency of solar energy utilization (Sun et al., 2018), leading to a significant increase in the photosynthetic rate. In addition, the milk stage is an important period of seed filling, which significantly increases water and mineral utilization and inevitably increases transpiration pull to provide sufficient water and nutrients to meet growth requirements. At the same time, the increased transpiration rate causes the leaf stomata to open, and airborne CO_2_ enters the leaf cells through the stomata, thereby increasing the intercellular CO_2_ concentration. The highest net photosynthetic rate, transpiration rate, and intercellular CO_2_ concentration were observed during the milk stage season. This is consistent with some reports (Liu et al., 2018). Water is a raw material for photosynthesis, and a higher water retention of leaves ensures a supply of photosynthetic water while maintaining cellular structure. The highest water retention in the leaves at the milk stage in this study was similar to that reported by Wu (Wu et al., 2023).

In this study, although the concentration of mancozeb had no effect on leaf photosynthetic properties, the low treatment had slightly better photosynthetic properties than did the CK, medium, and high treatments. Therefore, the low treatment significantly increased silage maize yield compared to the CK. However, a smaller effect on photosynthesis may be related to the nature and function of the pesticides. Mancozeb, a fungicide, is mainly used to regulate the adverse effects of fungal diseases on crops (Munkvold et al., 2001; Rejali et al., 2022); in particular, it is used to prevent and reduce the damage caused by pathogenic fungi to plants (Lai et al., 2016; Schneider et al., 2023). When fungal diseases are not the dominant factors affecting photosynthesis in maize, mancozeb has little effect on the photosynthetic properties of the crop. In addition, leaf photosynthetic properties are mainly affected by leaf area, available light energy, efficiency of light capture, and temperature (Ye et al., 2022; Zhang et al., 2018), and the use of fungicides does not directly affect leaf area or the efficiency of light capture. Therefore, changes in the concentration of mancozeb do not have a significant effect on photosynthetic properties.

Leaf structure plays an important role in the defense against external environmental disturbances and in maintaining plant health (Javelle et al., 2011; Zuch et al., 2022). Usually, the lower epidermis of the leaf blade is closer to the ground and has a relatively high humidity, which helps reduce water loss by transpiration (Revilla et al., 2016; Zuch et al., 2022). In contrast, the upper surface of the leaf blade is susceptible to transpirational water loss owing to the higher temperatures of sunlight radiation. Therefore, the lower stomatal density of the upper epidermis of leaves compared with that of the lower epidermis also plays a protective role. In the present study, the upper epidermal stomatal densities of silage maize were lower than those at the trumpet, milk, and dough stages to better protect their health. Notably, the stomatal density was significantly higher at the dough stage than at the big trumpet stage. This is closely related to physiological and metabolic activities during the reproductive stage of maize (Kong et al., 2021; Nielsen, 2002). Because stomata play an important role in regulating plant water utilization and carbon gain (Bertolino et al., 2019), plants may maintain their physiological activities under dry climatic conditions by decreasing stomatal size and increasing density, thereby controlling water evaporation and gas exchange.

Trichomes enhance the protective effect of the leaf epidermis and reduce the aggression of adverse external environments (Zuch et al., 2022). For example, trichomes enhance leaf defense against pests and diseases, and they reduce water loss by lowering transpiration rates (Kong et al., 2021; Moya-Raygoza, 2016). In the present study, the upper trichomes density was significantly larger than that of the lower trichomes densitys in both the milk and dough stages of maize leaves, which is inconsistent with previously reported results (Watts and Kariyat, 2021). This may be because epidermal hair density is affected by both species and the environment. For example, some plant leaves have higher trichomes densities under direct sunlight and reduced moisture (Wang et al., 2021b), and dense trichomes can regulate the heat balance and photon interception of leaves, which in turn affects gas exchange properties (Bickford, 2016). In this study, we found that cuticle permeability was significantly lower at the big trumpet and milk stages than at the dough stage (from 1 to 5 h). This is because the big trumpet and milk stages are periods of rapid maize growth; plant metabolism is vigorous and has a great demand for water, and the plant needs to reduce water dissipation to ensure normal physiological metabolism. In contrast, maize tends to mature during the dough stage, and metabolism is gradually weakened; therefore, the water demand is relatively reduced. In addition, as maize reaches physiological maturity, the wax content on the leaf surface increases, which reduces the water-loss rate of maize leaves during the dough stage to a certain extent. In this study, changes in mancozeb concentration had no significant effect on stomatal and trichomes densities, or cuticle permeability of maize leaves. This is because the structural properties of maize leaves are affected more by the environment and its own multiple factors. When the pathogen is not the dominant factor affecting the growth of leaves, the use of fungicides will not have a significant effect on the structural properties of leaves.

### Effect of mancozeb concentration and maturity stage on leaf biochemical properties

During dough, the rate of nutrient translocation from leaves to kernels of maize decreases, but the chlorophyll contained in leaves (Elos et al., 2016) is still photosynthesized, and this synthesized organic matter is stored in leaves rather than transported to kernels. Therefore, the total phenol, proline, reducing sugar, vitamin C, free amino acid, and inorganic phosphorus contents of the leaves increases accordingly. During maize maturation, polysaccharides such as starch are converted into reducing sugars to meet the energy requirements for kernel maturation. In addition, temperatures are significantly lower at the dough stage, which reduces the rate of organic matter transport from the leaves to kernels. In turn, the higher levels of reducing sugars in the leaves in this study may have made the plants more adaptable to the adverse effects of lower temperatures. Similar results have been reported in several studies (Vágújfalvi et al., 1999). Phenolic are plant secondary metabolites with antioxidant properties that have potential health benefits for plants (Randhir and Shetty, 2005), low temperatures promote the synthesis of phenolic compounds and their subsequent adulteration into the plant cell wall as lignin or corky lipids (Ahlawat et al., 2024). In the present study, the total phenol content in silage maize leaves was the highest at the dough stage. In addition to the above-mentioned factors, the low-temperature environment faced by maize at the dough stage was also important.

Usually, the aboveground phosphorus concentration in crops decreases with increasing fertility stage (Ning et al., 2013). In this study, silage maize had the highest phosphoru content at the dough stage, which was not consistent with previous studies (Ning et al., 2013), and which may have been influenced by changes in plant root uptake capacity. Maize enters the dough stage once the leaves are not sufficient for obtaining nutrients, but the root system may activate a compensatory mechanism to accelerate nutrient uptake to maintain normal physiological function, which increases the phosphorus content in the leaves. Previous studies have also confirmed that phosphoru accumulate when leaves wither (Huang et al., 2020). In plants, proline accumulates in response to environmental stress (Szabados and Savouré, 2010). In the present study, the highest proline content was found in silage maize leaves at the dough stage because low temperature and water stress at dough affect proline accumulation in plants (Carceller and FrasChina, 1980; Duncan and Widholm, 1987). Vitamin C, an antioxidant and redox buffer, is important in the plant’s response to abiotic stresses and pathogens (Ishikawa et al., 2006), including the protection of plant cells against a number of induced oxidative stresses (Boubakri, 2017), and the enhancement of plant defense against pathogens (Barth et al., 2006). In the present study, the vitamin C content in silage maize leaves was the highest at the dough stage, which may have been due to a protective mechanism triggered by silage maize in response to leaf senescence. In the present study, the changes in free amino acid and protein content in silage maize leaves at the dough stage were similar to those of previous studies (Osaki et al., 1991). As silage maize matures, proteins in the leaves are broken down into free amino acids, and these amino acids are translocated to the harvested organs.

### Effect of mancozeb concentration and maturity stage on microbial survival of silage maize leaves

The big trumpet stage is an important stage in the transition from nutritive to reproductive growth in maize, when vigorous vital activities of the plant may lead to increased leaf secretion. For example, nutrients such as sugars, amino acids, organic acids, and a few minerals spill over from the leaf interior to the surface (Fatima and Senthil-Kumar, 2015), providing an important source of nutrients for microbial colonization. In addition, the big trumpet and milk stages occur in the warm and humid season, and the moist environment on the leaf surface facilitates microbial colonization and growth (Aung et al., 2018; Wu et al., 2023). In contrast, maize tends to mature during the dough stage, and dryness and reduced secretions from the leaf surface are unfavorable for microbial survival. In this study, the number of microorganisms was greater at both the trumpet and milk maturity stages than at the dough stage (**Supplementary Table S1**), and changes in nutrients, temperature, and humidity on the surface of the maize leaves were one of the reasons for this change.

Generally, beneficial plant interleaf microorganisms are unfavorable for the survival and multiplication of various plant pathogens (Chaudhry et al., 2021). They reduce pathogen infestation and plant diseases through competitive inhibition (Du et al., 2024), the production of antibiotics (Gerardin et al., 2016), and activation of plant defense systems (Zhu et al., 2022). Similarly, when plants are subjected to unfavorable external environments, the abundance of beneficial interleaf microorganisms increases to enhance defense mechanisms against environmental stress (Ali et al., 2024; Liu et al., 2020). In the present study, the use of mancozeb increased the relative abundance of the beneficial bacteria *Microbacterium* (**Table 4**). Similarly, low and medium treatments increased the relative abundance of the beneficial bacteria *Chryseobacterium* and *Massilia* (**Figure 3B**). This suggests that the use of mancozeb promotes the interleaf colonization of maize by some beneficial bacteria to varying degrees, which is important for silage maize to reduce the growth of plant pathogens and withstand environmental stresses. It was found that the establishment of certain plant microbial populations affects the survival and reproduction of other microbes, and that some microbes preferentially seize ecological niches and compete for nutrients (Wang et al., 2021a), leading to a decrease in the abundance of others, which is not an exception for plant pathogens and beneficial bacteria. The reasons for this are overlapping ecological niches and limited nutrient resources (Wang et al., 2021a). In view of these reasons, in the present study, the medium and high treatments bacterium *Sphingomonas* behaved as a dominant bacterium and was beneficial to plants (**Figure 3**; **Table 4**), which may have led to a decrease in the relative abundance of bacteria such as *Comamonadaceae*, and *Stenotrophomonas*. In the present study, it was found that low, medium and high treatments shared a certain number of bacterial OTUs with the CK treatment. The resistance of some of the bacteria (Gondal et al., 2012; Islam et al., 2024) also contributed to this result, which was able to survive even with the change in concentration. There were differences in the effects of mancozeb concentration on the bacterial community. Specifically, the number of bacterial OTUs was higher in the low treatment than in the CK, and lower in the medium and high treatments than in the CK. Mancozeb is a broad-spectrum fungicide that can negatively affect or even inhibit the growth of bacteria on leaves (Huang et al., 2021; Vuyyuru et al., 2018). For example, in a sugarcane inter-root microbial study, mancozeb was found to reduce the number of bacteria and fungi (Vuyyuru et al., 2018). Medium and high treatments under high selective pressure are unfavorable for bacterial survival, leading to a reduction in bacterial OTU. The limited effect of low treatment on bacterial growth inhibition and the susceptibility of some microorganisms to developing resistance (Gondal et al., 2012) led to the survival and multiplication of this treatment of microorganisms, which generally increased the diversity of bacterial OTUs.

In terms of biodiversity and plant health, the interaction between multiple microorganisms (Tharanath et al., 2024) is favorable for increasing the adaptability of plants to the environment to ensure plant health. In the present study, medium and low treatments reduced the relative abundance of harmful fungi such as *Gibberella* and *Epicoccum* (**Table 4**; **Figure 4B**). This resulted from the direct action of the fungicide mancozeb (Liu et al., 2015) or the use of mancozeb to promote the growth and multiplication of beneficial fungi such as *Filobasidium*, *Vishniacozyma*, *Papiliotrema* and other fungi (**Figure 4B**) in maize interfoliage, which resulted in the inhibition of the colonization of harmful fungi. On the other hand, mancozeb treatment increased fungal OTU counts. Although there is no direct evidence that mancozeb pronounced inhibits the population of dominant fungal communities on maize leaves, it is possible that as a fungicide it reduces the population of dominant fungi on maize leaves to some extent. As a result, despite the limited nutrients on maize leaves, it is still able to support the growth and reproduction of some minor fungi (Karlsson et al., 2014), resulting in increased fungal diversity. Overall, the use of mancozeb with varying degrees of low promotes the survival and reproduction of beneficial leaf microorganisms and reduces the colonization and damage of plant pathogens in maize leaves.

## Conclusion

Based on the yield and utilization of silage maize, and considering the changes in the diversity of microorganisms adhering to the surface of silage maize leaves, this study recommends the use of low concentrations of Mancozeb and harvesting at the dough stage.

## Data Availability

The raw data supporting the conclusions of this article will be made available by the authors, without undue reservation.
