# Supplementary figures and images for: Effects of maturity stage and mancozeb on phyllosphere microbial communities and the plant health potential of silage maize
Source: Front Plant Sci. 2025 Jun 4;16:1581401. doi: 10.3389/fpls.2025.1581401 (PMC12174089; doi:10.3389/fpls.2025.1581401)

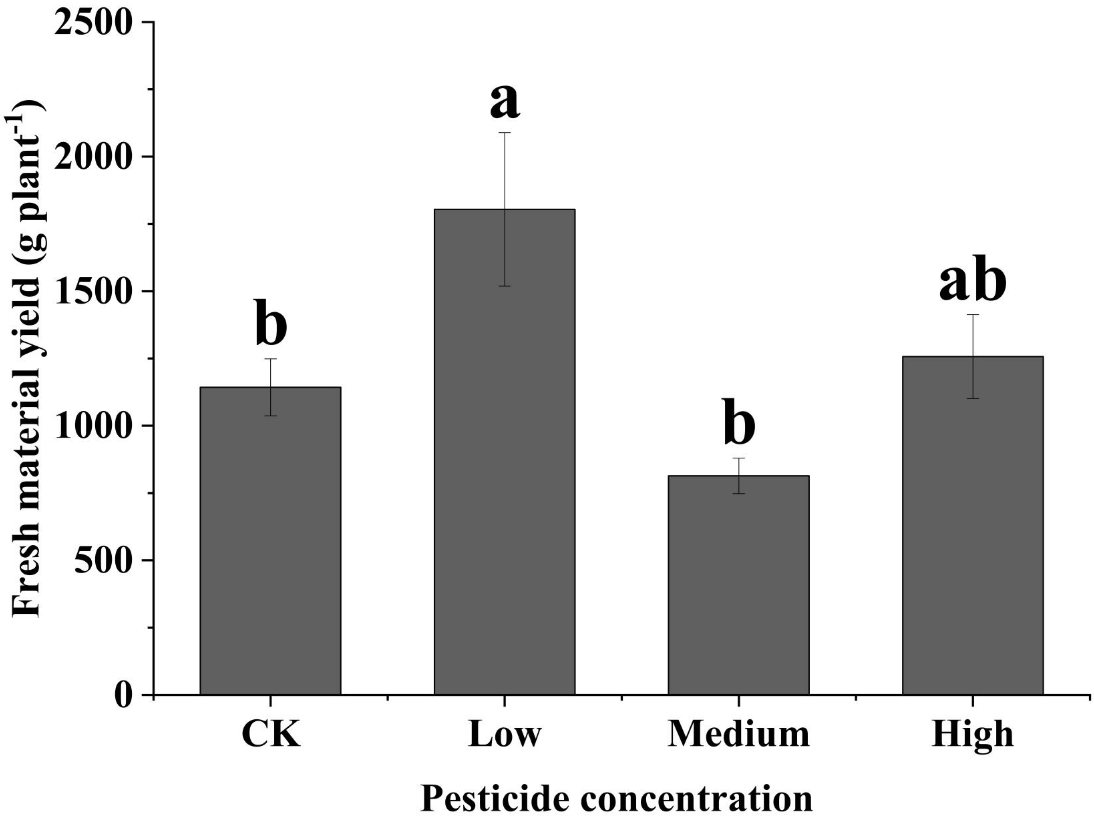

Supplement: Supplementary file 1 [file Image1.jpeg]
