# Supplementary material for: Effects of maturity stage and mancozeb on phyllosphere microbial communities and the plant health potential of silage maize
Source: Front Plant Sci. 2025 Jun 4;16:1581401. doi: 10.3389/fpls.2025.1581401 (PMC12174089; doi:10.3389/fpls.2025.1581401)
Supplement: Supplementary file 2 [file Table1.docx]

Table S 1 Effects of maturity stage and pesticide concentration on microorganisms numbers of silage corn leaves (n = 12)

| Maturity  stage and treatment | | Aerobic bacteria (lg cfu g⁻¹ FM) | *Escherichia coli*  (lg cfu g⁻¹ FM) | Yeast  (lg cfu g⁻¹ FM) | Molds  (lg cfu g⁻¹ FM) | Lactic acid bacteria  (lg cfu g⁻¹ FM) |
| --- | --- | --- | --- | --- | --- | --- |
| Maturity stage (MS) | Big trumpet stage | 7.42a | 6.88a | 6.14a | 4.86a | 4.78a |
|  | Milk stage | 5.18b | 5.32b | 4.90b | 4.09b | 4.42a |
|  | Dough stage | 4.06b | 4.40c | 4.31c | 3.36b | 2.69 |
|  | Average value | 5.73 | 5.53 | 5.12 | 4.1 | 3.96b |
|  | Quadratic sum | 53.2 | 37.8 | 20.9 | 13.5 | 29.8 |
|  | *F* | 34.2 | 20.6 | 22.1 | 8.4 | 16.9 |
| Pesticide concentration (PE) | CK | 5.21 | 4.68 | 4.75 | 3.81 | 3.14 |
|  | Low | 5.79 | 6.05 | 5.31 | 4.23 | 4.45 |
|  | Medium | 6.03 | 5.53 | 5.45 | 4.3 | 4.2 |
|  | High | 5.92 | 5.86 | 4.96 | 4.07 | 4.07 |
|  | Average value | 5.74 | 5.53 | 5.12 | 4.1 | 3.96 |
|  | Quadratic sum | 3.62 | 10 | 2.75 | 1.26 | 8.81 |
|  | *F* | 0.51 | 1.85 | 0.87 | 0.35 | 1.87 |
| *P* value | MS | 0 | 0 | 0 | 0.001 | 0 |
|  | PE | 0.676 | 0.158 | 0.469 | 0.792 | 0.154 |
|  | MS×PE | 0.006 | 0.003 | 0.019 | 0.592 | 0 |
|  | Quadratic sum (MS×PE) | 11 | 10.9 | 5.71 | 4.15 | 12.5 |
|  | *F* (MS×PE) | 4.01 | 4.7 | 3.18 | 0.78 | 6.36 |
| SEM | | 0.25 | 0.23 | 0.17 | 0.18 | 0.22 |

Note: Different lowercase letters in the same column represent significant difference between maturity stage or pesticide concentrations (P < 0.05). SEM, standard errors of the mean.
